# Supplementary material for: Heat adaptation of phage T7 under an extended genetic code
Source: Virus Evol. 2021 Dec 1;7(2):veab100. doi: 10.1093/ve/veab100 (PMC8923235; doi:10.1093/ve/veab100)
Supplement: veab100_Supp [file veab100_supp.zip › Sequencing_Analysis.zip!/Sequencing_Analysis/S3/evidence/SNP_13.html]

BRESEQ :: Evidence


| Predicted mutation | | | | | | |
| --- | --- | --- | --- | --- | --- | --- |
| evidence | seq id | position | mutation | annotation | gene | description |
| RA | NC\_001604.1 | 30,869 | G→A | intergenic (–/–) | – / – | –/– |

| Read alignment evidence... | | | | | | | | | | | |
| --- | --- | --- | --- | --- | --- | --- | --- | --- | --- | --- | --- |
|  | seq id | position | | ref | new | freq | score (cons/poly) | reads | annotation | genes | product |
| \* | NC\_001604.1 | 30,869 | 0 | G | A | 88.8% | 3909.3 / inf | 1766 | intergenic (–/–) | *–/–* | –/– |
| Reads supporting (aligned to +/- strand):  **ref** base G (88/108);  **new** base A (779/777);  **total** (879/887) | | | | | | | | | | | |
| Fisher's exact test for biased strand distribution *p*-value = 1.97e-01 | | | | | | | | | | | |
| Kolmogorov-Smirnov test that lower quality scores support variant *p*-value = 1.43e-01 | | | | | | | | | | | |

Only 100 of 1769 total aligned reads displayed.  

`AAAGGCTACATAAGGAGGCCCTAAATGGATAAGTACGATAAGAACGTACCAAGTGATTATGATGGTCTGTTCCAAAAGGCTGCTGATGCCAACGGGGTCTCTTATGACCTTTTACGTAAAGTCGCTTGGACAGAATCACGATTTGTGCCTACAGCAAAATCTAAGACTGGACCATTAGGCATGATGCAATTTACCAAGGCAACCGCTAAGGCCCTCGGTCTGCGAGTTACCGATGGTCCAGACGACGACCGACTGAACCCTGAGTTAGCTATTAATGCTGCCGCTAAGCAACTTGCAGGTCTGGTAGGGAAGTTTGATGGCGATGAACTCAAAGCTGCCCTTGCGTACAACCAAGGCGAGGGACGCTTGGGTAATCCACAACTTGAGGCGTACTCTAAGGGAGACTTCGCATCAATCTCTGAGGAGGGACGTAACTACATGCGTAACCTTCTGGATGTTGCTAAGTCACCTATGGCTGGACAGTTGGAAACTTTTGGTGGCATAACCCCAAAGGGTAAAGGCATTCCGGCTGAGGTAGGATTGGCTGGAATTGGTCACAAGCAGAAAGTAACACAGGAACTTCCTGAGTCCACAAG  >  NC_001604.1/30571‑31166`  

                                                                                                                                                                                                                                                                                                          `|`                                                                                                                                                                                                                                                                                                           
   `ggCTACATAAGGAGGCCCTAAATGGATAAGTACGATAAGAACGTACCAAGTGATTATGATGGTCTGTTCCAAAAGGCTGCTGATGCCAACGGGGTCTCTTATGACCTTTTACGTAAAGTCGCTTGGACAGAATCACGATTTGTGCCTACAGCAAAATCTAAGACTGGACCATTAGGCATGATGCAATTTACCAAGGCAACCGCTAAGGCCCTCGGTCTGCGAGTTACCGATGGTCCAGACGACGACCGACTGAACCCTGAGTTAGCTATTAATGCTGCCGCTAAGCAACTTGCAGATCTgg                                                                                                                                                                                                                                                                                                      <  1:207245/301‑1 (MQ=255)`  
   `ggCTACATAAGGAGGCCCTAAATGGATAAGTACGATAAGAACGTACCAAGTGATTATGATGGTCTGTTCCAAAAGGCTGCTGATGCCAACGGGGTCTCTTATGACCTTTTACGTAAAGTAGCTTGGACAGAATCACGATTTGTGCCTACAGCAAAATCTAAGACTGGACCATTAGGCATGATGCAATTTACCAAGGCAACCGCTAAGGCCCTCGGTCTGCGAGTTACCGATGGTCCAGACGACGACCGACTGAACCCTGAGTTAGCTATTAATGCTGCCGCTAAGCAACTTGCAGATCTgg                                                                                                                                                                                                                                                                                                      >  1:159557/1‑301 (MQ=255)`  
     `cTACATAAGGAGGCCCTAAATGGATAAGTACGATAAGAACGTACCAAGTGATTATGATGGTCTGTTCCAAAAGGCTGCTGATGCCAACGGGGTCTCTTATGACCTTTTACGTAAAGTAGCTTGGACAGAATCACGATTTGTGCCTACAGCAAAATCTAAGACTGGACCATTAGGCATGATGCAATTTACCAAGGCAACCGCAAAGGCCCTCGGTATGCGAGTTACCGATGGTCCAGACGACGACCGACTGAACCCTGAGTTAGCTATTCATGCTGCCGCTAAGCAACTTGCAGATCTGGTa                                                                                                                                                                                                                                                                                                    >  1:333594/1‑301 (MQ=255)`  
       `aCATAAGGAGGCCCTAAATGGATAAGTACGATAAGAACGTACCAAGTGATTATGATGGTCTGTTCCAAAAGGCTGCTGATGCCAACGGGGTCTCTTATGACCTTTTACGTAAAGTCGCTTGGACAGAATCGCGATTTGTGCCTACAGCAAAATCTAAGACTGGACCATTAGGCATGATGCAATTTACCAAGGCAACCGCTAAGGCCCTCGGTCTGCGAGTTACCGATGGTCCAGACGACGACCGACTGAACCCTGAGTTAGCTATTAATGCTGCCGCTAAGCAACTTGCAGATCTGGTCgg                                                                                                                                                                                                                                                                                                  >  1:328270/1‑301 (MQ=255)`  
       `aCATAAGGAGGCCCTAAATGGATAAGTACGATAAGAACGTACCAAGTGATTATGATGGTCTGTTCCAAAAGGCTGCTGATGCCAACGGGGTCTCTTATGACCTTTTACGTAAAGTCGCTTGGACAGAATCACGATTTGTGCCTACAGCAAAATCTAAGACTGGACCATTAGGCATGATGCAATTTACCAAGGCAACCGCTAAGGCCCTCGGTCTGCGAGTTACCGATGGTCCAGACGACGACCGACTGAACCCTGAGTTAGCTATTAATGCTGCCGCTAAGCAACTTGCAGGTCTGGTAgg                                                                                                                                                                                                                                                                                                  >  1:72298/1‑301 (MQ=255)`  
        `cATCAGGAGGCCCTAAATGGATAAGTACGATAAGAACGTACCAAGTGATTATGATGGTCTGTTCCAAAAGGCTGCTGATGCCAACGGGGTCTCTTATGACCTTTTACGTAAAGTCGCTTGGACAGAATCACGATTTGTGCCTACAGCAAAATCTAAGACTGGACCCTTAGTCATGATGCAATTTACCAAGGCAACCGCTAAGGCCCTCGGTCTGCGAGTTACCGATGGTCCAGACGACGACCGACTGAACCCTGAGTTACCTATTAATGCTGCCGATAAGCAACTTGCAGATCTGTCAggg                                                                                                                                                                                                                                                                                                 >  1:210753/1‑301 (MQ=255)`  
        `aaTAAGGAGGCCCTAAATGGATAAGTACGATAAGAACGTACCAAGTGATTATGATGGTCTGTTCCAAAAGGCTGCTGATGCCAACGGGGTCTCTTATGACCTTTTACGTAAAGTCGCTTGGACAGAATCACGATTTGTGCCTACAGCAAAATCTAAGACTGGACCATTAGGCATGATGCAATTTACCAAGGCAACCGCTAAGGCCCTCGGTCTGCGAGTTACCGATGGTCCAGACGACGACCGACTGAACCCTGAGTTAGCTATTAATGCTGCCGCTAAGCAACTTGCAGATCTGGTAggg                                                                                                                                                                                                                                                                                                 <  1:78049/300‑1 (MQ=255)`  
           `aaGGATGCCCTAAAGGGATAAGTAAGATAAGAACGTACCAAGTGATTAGGATGGTCTGTTCCAAAAGGCTGCTGATGCCAACGGGTTATCTTATGACCTTTTAAGTAAAGTCGATTGGACAGAATCACGATTTGTGCCTACAGCAAAATCTAAGACTGGACCATTAGGCATGATGCAATTTACCAAGGCAACCGCTAAGGCCCTCGGTCTGCGAGTTACCGATGGTCCAGACGACGACCGACTGAACCCTGAGTTAGCTATTAATGCTGCCGCTAAGCAACTTGCAGGTCTGGTAGGGAAg                                                                                                                                                                                                                                                                                              <  1:109444/301‑1 (MQ=255)`  
                 `gCCCTAAATGGATCAGTACGATAAGAACGTACCAAGTGATTATGATGGTCTGTTCCAAAAGGCTGCTGATGCCAACGGGGTCTCTTATGACCTTTTACGTAAAGTCGCTTGGACAGAATCACGATTTGTGCCTACAGCAAAATCTAAGACTGGACCATTAGGCATGATGCAATTTACTAAGGCAACCGCTAAGGCCCTCGGTCTGCGAGTTACCGATCGTCCAGACGTCGACCGACTGAACCCTGAGTTAGCTATTAATGCTGCCGCTAAGCCACTTGCAGACCTGTTAGGGAAGCTTGAt                                                                                                                                                                                                                                                                                        >  1:50257/1‑301 (MQ=255)`  
                   `agggacaggATAAGTACGATAAGAACGTACCAAGTGATTATGATGGTCTGTTCCAAAAGGCTGCTGATGCCAACGGGGTCTCTTATGACCTTTTACGTAAAGTCGCTTTGACAGAATCACGATTTGTGCCTACAGCAAAATCTAAGACTGGACCATTAGGCATGATGCAATTTACCAAGGCAACCGCGAAGTCCCTCGGTCTGCGAGTTACCGATGGTCCAGACGACGACCGACTGAACCCTGAGTTAGCTATTAATGCTGCCGCTAAGCAACTTGCAGATCTGGTAGGGAAGTTTGATgg                                                                                                                                                                                                                                                                                      <  1:268872/294‑1 (MQ=255)`  
                           `gATAAGTACGATAAGAACGTACCAAGTGATTATGATGGTCTGTTCCAAAAGGCTGCTGATGCCAACGGGGTCTCTTATGACCTTTTACGTAAAGTCGCTTGGACAGAATCACGATTTGTGCCTACAGCAAAATCTAAGACTGGACCATTAGGCATGATGCAATTTATCAAGGCAACCGCTAAGGCCCTCGGTCTGCGAGTTACCGATGGTCCAGACGACGACCGACTGAACCCTGAGTTAGCTATTAATGCTGCCGCTAAGCAACTTGCAGATCTGGTAGGGAAGTTTGATGGCGATGAAc                                                                                                                                                                                                                                                                              >  1:115030/1‑301 (MQ=255)`  
                           `gATAAGTACGATAAGAACGTACCAAGTGATTATGATGGTCTGTTCCAAAAGGCTGCTGATGCCAACGGGGTCTCTTATGACCTTTTACGTAAAGTCGCTTGGACAGAATCACGATTTGTGCCTACAGCAAAATCTAAGACTGGACCATTAGGCATGATGCAATTTACCAAGGCAACCGCTAAGGCCCTCGGTCTGCGAGTTACCGATGGTCCAGACGACGACCGACTGAACCCTGAGTTAGCGATTAATGCTGCCGATAAGCAACTTGCAGATCTGGTAGGGAAGTTTGATGGGGATGaaa                                                                                                                                                                                                                                                                              >  1:296321/1‑300 (MQ=255)`  
                                   `cGATAAGAACGTACCAAGTGATTATGATGGTCTGTTCCAAAAGGCTGCTGATGCCAACGGGGTCTCTTATGACCTTTTACGTAAAGTCGCTTGGACAGAATCACGATTTGTGCCTACAGCAAAATCTAAGACTGGACCATTAGGCATGATGCAATTTACCAAGGCAACTGCTAAGGCCCTCGGTCTGCGAGTTACCGATGGTCCAGACGACGACCGACTGAACCCTGAGTTAGCTATTAATGCTGCCGCTAAGCAACTTGCAGATCTGGTAGGGAAGTTTGATGGCGATGAACTCAAAGCt                                                                                                                                                                                                                                                                      >  1:53805/1‑301 (MQ=255)`  
                                   `cGATAAGAACGTACCAAGTGATTATGATGGTCTGTTCCAAAAGGCTGCTGATGCCAACGGGGTCTCTTATGACCTTTTACGTAAAGTCGCTTGGACAGAATCACGATTTGTGCCTACAGCAAAATCTAAGACTGGACCATTAGGCATGATGCAATTTACCAAGGCAACCGCTAAGGCCCTCGGTCTGCGAGTTACCGATGGTCCAGACGACGACCGACTGAACCCTGAGTTAGCTATTAATGCTGCCGCTAAGCAACTTGCAGGTCTGGTAGGGAAGTTTGATGGCGATGAACTCAAAGCt                                                                                                                                                                                                                                                                      >  1:250212/1‑301 (MQ=255)`  
                                   `cGATAAGAACGTACCAAGTGATTATGATGGTCTGTTCCAAAAGGCTGCTGATGCCAACGGGGTCTCTTATGACCTTTTACGTAAAGTCGCTCGGACAGAATCACGATTGGTGCCTACAGCAAAATCTAAGACTGGACCATTAGGCATGATGCAATTTACCAAGGCAACCGCTAAGGCCCTCGGTCTGCGAGTTACCGATGGTCCAGACGACGACCGACTGAACCCTGAGTTAGCCATTAATGCTGCCGCTAAGCAACTTGCAGATCTGGTAGGGAAGTTTGATGGCTATGAACTCCAAGcc                                                                                                                                                                                                                                                                      >  1:192469/1‑300 (MQ=255)`  
                                     `aTAAGAACGTACCAAGTGATTATGATGGTCTGTTCCAAAAGGCTGCTGATGCCAACGGGGTCTCTTATGACCTTTTACGTAAAGTCGCTTGGACAGAATCACGATTTGTGCCTACAGCAAAATCTAAGACTGGACCATTAGGCATGATGCAATTTACCAAGGCAACCGCTAAGGCCCTCGGTCTGCGAGTTACCGATGGTCCAGACGACGACCGACTGAACCCTGAGTTAGCTATTAATGCTGCCGCTAAGCAACTTGCAGATCTGGTAGGGAAGTTTGATGGCGATGAACTCAAAGCTGc                                                                                                                                                                                                                                                                    >  1:80144/1‑301 (MQ=255)`  
                                      `tAAGAACGTACCAAGTGATTATGATGGTCTGTTCCAAAAGGCTGCTGATGCCAACGGGGTCTCTTATGACCTTTTACGTAAAGTCGCTTGGACAGAATCACGATTTGTGCCTACAGCAAAATCTAAGACTGGACCATTAGGCATGATGCAATTTACCAAGGCAACCGCTAAGGCCCTCGGTCTGCGAGTTACCGATGGTCCAGACGACGACCGACTGAACCCTGAGTTAGCTATTAATGCTGCCGCTAAGCAACTTGCAGATCTGGTAGGGAAGTTTGATGGCGATGAACTCAAAGCTGcc                                                                                                                                                                                                                                                                   <  1:266120/301‑1 (MQ=255)`  
                                        `aGAACGTACCAAGTGATTATGATGGTCTGTTCCAAAAGGCTGCTGATGCCAACGGGGTCTCTTATGACCTTTTACGTAAAGTCGCTTGGACAGAATCACGATTTGTGCCTACAGCAAAATCTAAGACTGGACCATTAGGCATGATGCAATTTACCAAGGCAACCGCTAAGGCCCTCGGTCTGCGAGTTACCGATGGTCCAGACGACGACCGACTGAACCCTGAGTTAGCTATTAATGCTGCCGCTAAGCAACTTGCAGATCTGGTAGGGAAGTTTGATGGCGATGAACTCAAAGCTGCCCt                                                                                                                                                                                                                                                                 >  1:307380/1‑301 (MQ=255)`  
                                            `cGTACCAAGTGATTATGATGGTCTGTTCCAAAAGGCTGCTGATGCCAACGGGGTCTCTTATGACCTTTTACGTAAAGTCGCTTGGACAGAATCACGATTTGTGCCTACAGCAAAATCTAAGACTGGACCATTAGGCATGATGCAATTTACCAAGGCAACCGCTAAGGCCCTCGGTCTGCGAGTTACCGATGGTCCAGACGACGACCGACTTAACCCTGAGTTAGCTATTAATGCTGCCGCTAAGCAACTTGCAGGTCTGGTAGGGAAGTTTGATGGCGATGAACTCAAAGCTGCCCTTGCg                                                                                                                                                                                                                                                             <  1:173722/301‑1 (MQ=255)`  
                                            `cGTACCAAGTGATTATGATGGTCTGTTCCAAAAGGCTGCTGATGCCAACGGGGTCTCTTATGACCTTTTACGTAAAGTCGCTTGGACAGAATCACGATTTGTGCCTACAGCAAAATCTAAGACTGGACCATTAGGCATGATGCAATTTACCAAGGCAACCGCTAAGGCCCTCGGTCTGCGAGTTACCGATGGTCCAGACGACGACCGACTGAACCCTGAGTTAGCTATTAATGCTGCCGCTAAGCAACTTGCAGATCTGGTAGGGAAGTTTGATGGCGATGAACTCAAAGCTGCCCTTGCg                                                                                                                                                                                                                                                             >  1:2467/1‑301 (MQ=255)`  
                                             `gTACCAAGTGATTATGATGGTCTGTTCCAAAAGGCTGCTGATGCCAACGGGGTCTCTTATGACCTTTTACGTAAAGTCGCTTGGACAGAATCACGATTTGTGCCTACAGCAAAATCTAAGACTGGACCATTAGGCATGATGCAATTTACCAAGGCAACCGCTAAGGCCCTCGGTCTGCGAGTTACCGATGGTCCAGACGACGACCGACTGAACCCTGAGTTAGCTATAAATGCTGCCGCTAAGCAACTTGCAGGTCTGGTAGGGAAGTTTGATGGCGATGAACTCAAAGCTGCCCTTGCGt                                                                                                                                                                                                                                                            <  1:284035/301‑1 (MQ=255)`  
                                              `tACCAAGTGATTATGATGGTCTGTTCCAAAAGGCTGCTGATGCCAACGGGGTCTCTTATGAACTTTTACGTAAAGTCGCTTGGACAGAATCACGATTTGTGCCTACAGCAAAATCTAAGACTGGACCATTAGGCATGATGCAATTTACCAAGTCAACCGCTAAGGCCCTCGGTCTGCGAGTTACCGATGGTCCAGACGACGACCGACTGAACCCTGAGTTAGCTATTAATGCTGCCGCTAAGCAACTTGCAGATCTGGTAGGGAAGTTTGATGGCGATGAACTCAAAGCTGCACTTGAGTa                                                                                                                                                                                                                                                           >  1:180299/1‑301 (MQ=255)`  
                                               `cagaaGTGATTATGATGGTCTGTTCCAAAAGGCTGCTGATGCCAACGGGGTCTCTTATGACCTTTTACGTAAAGTCGCTTGGACAGAATCACGATTTGTGCCTACAGCAAAATCTAAGACTGGACCATTATGCATGATGCAATTTACCAAGGCAACCGCTAAGGCCCTCGGTCTGCGAGTTACCGATGGTCCAGACGACGACCGACTGAACCCTGAGTTAGCTATTAATGCTGCCGCTAAGCAACTTGCAGATCTGGTAGGGAAGTTTGATGGCGATGAACTCAAAGCTGCCCTTGCGTAc                                                                                                                                                                                                                                                          <  1:239864/298‑1 (MQ=255)`  
                                               `agaagagacagatgaagGTCTGTTCCAAAAGGCTGCTGATGCCAACGGGGGATCTTATGACCTTTTACGTAAAGTCGCTTGGACAGAATCACAATTTGGGCCTACAGCAAAATCTAAGACTGGACCATTAGGCATGATGCAATTTACCAAGGCAACCGCTAAGGCCCTCAGTCTGCGAGTTACCGATGGTCCAGACGACGACCGACTGAACCCTGAGTTAGCTATTAATGCTGCAGATAAGCAACTTGCAGATCTGGTAGGGAAGTTTGATGGCGATGAACTCAAAGCTGCCCTTGCGTAc                                                                                                                                                                                                                                                          <  1:36814/290‑1 (MQ=255)`  
                                                   `agggATTATGATGGTCTGTTCCAAAAGGCTGCTGATGCCAACGGGGTCTCTTATGACCTTTTACGTAAAGTCGCTTGGACAGAATCACGATTTGTGCCTACAGCAAAATCTAAGACTGGACCATTAGGCATGATGCAATTTACCAAGGCAACCGCTAAGGCCCTCGGTCTGCGAGTTACCGATGGTCCAGACGACGACCGACTGAACCCTGAGTTAGCTATTAATGCTGCCGATAAGCAACTTGCAGATCTGGTAGGGAAGTTTGATGGCGATGAACTCAAAGCTGCCCTTGCGTACAAcc                                                                                                                                                                                                                                                      <  1:85273/298‑1 (MQ=255)`  
                                                    `gTGATTATGATGGTCTGTTCCAAAAGGCTGCTGATGCCAACGGGGTCTCTTATGACCTTTTACGTAAAGTCGCTTGGACAGAATCACGATTTGTGCCTACAGCAAAATCTAAGACTGGACCATTAGGCATGATGCAATTTACCAAGGCAACCGCTAAGGCCCTCGGTCTGCGAGTTACCGATGGTCCAGACGACGACCGACTGAACCCTGAGTTAGCTATTAATGCTGCCGCTAAGCAACTTGCAGATCTGGTAGGGAAGTTTGATGGCGATGAACTCAAAGCTGCCCTTGCGTACAACCa                                                                                                                                                                                                                                                     <  1:220806/301‑1 (MQ=255)`  
                                                      `gATTATGATGGTCTGTTCCAAAAGGCTGCTGATGCCAACGGGGTCTCTTATGACCTTTTACGTAAAGTCGCTTGGACAGAATCACGATTTGTGCCTACAGCAAAATCTAAGACTGGACCATTAGGCATGATGCAATTTACCAAGGCAACCGCTAAGGCCCTCGGTCTGCGAGTTACCGATGGTCCTGACGACGACCGACTGAACCCTGAGTTAGCTATTAATGCTGCCTCTAAGCAACTTGCAGGTCTGGTAGGGAAGTTTGATGGCGATGAACTCAAAGCTGCCCtctgtctcttataca                                                                                                                                                                                                                                                   >  1:259167/1‑287 (MQ=255)`  
                                                       `aTTATGCTGGTCTGTTCCAAAAGTCTGCTGATGCCCACGGGGTCTCTTATGACCTTTTACGTAAAGTCGCTTGGACAGAATCACGATTTGTGCCTACAGCAAAATCTAAGACTGGACCATTAGGCATGATGCAATTTACCAAGGCAACCGCTAAGGCCCTCGGTCTGCGAGTTACCGATGGTCCAGACGACGACCGACTGAACCCTGAGTTAGCTATTAATGCTGCCGCTAAGCAACTTGCAGATCTGGTAGGGAAGTTTGATGGCGATGAACTCAAAGCTGCCCTTGCGTACAACCAAgg                                                                                                                                                                                                                                                  <  1:10394/301‑1 (MQ=255)`  
                                                       `aTTATGATGGTCTGTGCCAAAAGGCTGCTGATGCCAACGTGGTCTCTTATGACCTTTTACGTAAAGTCGCTTGGACAGAAGCACGATTTGTGCCTACAGCAAAATCTAAGACTGGACCATTAGGCAGGATGCAATTTACCAAAGCAACCGCTAAGGCCCTCGGTCTGCGAGTTACCGATGTTCCAGACGACGACCGACTGAACCCTGAGTTAGCTATTAATGCTGCCGCTAAGCAACTTGCAGATCTGGTAGGGAAGTTTGATGGCGATGAACTCAAAGCTGCCCTTGCGTACAACCAAgg                                                                                                                                                                                                                                                  <  1:286389/301‑1 (MQ=255)`  
                                                        `ttATGATGGTCTGTTCCAAAAGGCTGCTGATGCCAACGGGGTCTCTTATGACCTTTTACGTAAAGTCGCTTGGACAGAATCACGATTTGTGCCTACAGCAAAATCTAAGACTGGACCATTAGGCATGATGCAATTTACCAAGGCAACCGCTAAGGCCCTCGGTCTGCGAGTTACCGATGGTCCAGACGACGACCGACTGAACCCTGAGTTAGCTATTAATGCTGCCGCTAAGCAACTTGCAGATCTGGTAGGGAAGTTTGATGGCGATGAACTCAAAGCTGCCCTTGCGTACAACCAAGGc                                                                                                                                                                                                                                                 >  1:117691/1‑301 (MQ=255)`  
                                                         `tATGATGGTCTGTTCCAAAAGGCTGCTGATGCCAACGGGGTCTCTTATGACCTTTTACGTAAAGTCGCTTGGACAGAATCACGATTTGTGCCTACAGCAAAATCTAAGACTGGACCATTAGGCATGATGCAATTTACCAAGGCAACCGCTAAGGCCCTCGGTCTGCGAGTTACCGATGGTCCAGACGACGACCGACTGAACCCTGAGTTAGCTATTAATGCTGCCGCTAAGCAACTTGCAGATCTGGTAGGGAAGTTTGATGGCGATGAACTCAAAGCTGCCCTTGCGTACAACCAAGGCg                                                                                                                                                                                                                                                <  1:137088/301‑1 (MQ=255)`  
                                                          `atgatgGTCTGTTCCAAAAGGCTGCTGATGCCAACGGGGTCTCTTATGACCTTTTACGTAAAGTCGCTTGGACAGAATCACGATTTGTGCCTACAGCAAAATCTAAGACTGGACCATTAGGCATGATGCAATTTACCAAGGCAACCGCTAAGGCCCTCGGTCTGCGAGTTACCGATGGTCCAGACGACGACCGACTGAACCCTGAGTTAGCTATTAATGCTGCCGCTAAGCAACTTGCAGATCTGGTAGGGAAGTTTGATGGCGATGAACTCAAAGCTGCCCTTGCGTACAACCAATGCGa                                                                                                                                                                                                                                               >  1:87446/1‑301 (MQ=255)`  
                                                          `atgatgGTCTGTTCCAAAAGGCTGCTGATGCCAACGGGGTCTCTTATGACCTTTTACGTAAAGTCGCTTGGACAGAATCACGATTTGTGCCTACAGCAAAATCTAAGACTGGACCATTAGGCATGATGCAATTTACCAAGGCAACCGCTAAGGCCCTCGGTCTGCGAGTTACCGATGGTCCAGACGACGACCGACTGAACCCTGAGTTAGCTATTAATGCTGCCGCTAAGCAACTTGCAGATCTGGTAGGGAAGTTTGATGGCGATGAACTCAAAGCTGCCCTTGCGTACAACCAAGCCgg                                                                                                                                                                                                                                               >  1:276901/1‑300 (MQ=255)`  
                                                          `atgatgGTCTGTTCCAAAAGGCTGCTGATGCCAACGGGGTCTCTTATGACCTTTTACGTAAAGTCGCTTGGACAGAATCACGATTTGTGCCTACAGAAAAATCTAAGACTGGACCATTAGGCATGATGCAATTTACCAAGGCAACCGCTAAGGCCCTCGGTCTGCGAGTTACCGATGGTCCAGACGACGACCGACTGAACCCTGAGTTAGCTATTAATGCTGCCGCTAAGCAACTTGCAGATCTGGTAGGGAAGTTTGATGGCGATGAACTCAAAGCTGCCCTTGCGTACAAcctgtctct                                                                                                                                                                                                                                               >  1:199074/1‑294 (MQ=255)`  
                                                           `tgatgGTCTGTTCCAAAAGGCTGCTGATGCCAACGGGGTCTCTTATGACCTTTTACGTAAAGTCGCTTGGACAGAATCACGATTTGTGCCTACAGCAAAATCTAAGACTGGACCATTAGGCATGATGCAATTTACCAAGGCAACCGCTAAGGCCCTCGGTCTGCGAGTTACCGATGGTCCAGACGACGACCGACTGAACCCTGAGTTAGCTATTAATGCTGCCGCTAAGCAACTTGCAGATCTGGTAGGGAAGTTTGATGGCGATGAACTCAAAGCTGCCCTTGCGTACAACCAAGGCGAg                                                                                                                                                                                                                                              <  1:223312/301‑1 (MQ=255)`  
                                                              `tgGTCTGTTCCAAAAGGCTGCTGATGCCAACGGGGTCTCTTATGACCTTTTACGTAAAGTCGCTTGAACAGAATAACGATTTGTGTCTACAGCAAAATCAAAGACTGGACCATTAGGCATGATGCAATTTACCAAGGCAGCCGCTAAGACCCTCGGTCTGCGAGTTACCGATGGTCCAGACGACGACCGACTGAACCCTGAGTTAGCTATTAATGCTGCCGCTAAGCAACTTGCAGGTCTGGTAGGGAAGTTTGATGGCGATGAACTCAAAGCTGCCCTTGCGTACAACCAAGGCGAGGGa                                                                                                                                                                                                                                           <  1:130138/301‑1 (MQ=255)`  
                                                               `ggTCTGTTCCAAAAGGCTGCTGATGCCAACGGGGTCTCTTATGACCTTTTACGTAAAGTCGCTTGGACAGATTCACGTTTTGTGCCTACTGCAAAATCTTAGACTTGACCATTTCTCTTCATGCAATTTACCTTGGCAACCTCTACTTCCCTCGGTCTGCTAGTTACCGATGTTCCAGACGACGACCTACTGAACCCTGAGTTAGCTTTTATTGCTGCCGCTAAGCTTCTTTCAGGCTTGTTCTGGTAGTTTGTTGGCGATGTACTCAAAGCTGCCCTTTCGTTCAACCAAGGCgtgctcc                                                                                                                                                                                                                                          >  1:111640/1‑295 (MQ=255)`  
                                                                    `gTTCCAAAAGGCTGCTGATGCCAACGGGGTCTCTTATGACCTTTTACGTAAAGTCGCTTGGACAGAATCACGATTTGTGCCTACAGCAAAATCTAAGACTGGACCATTAGGCATTATGCAATTTACCAAGGCAACCGCTAAGGCCCTCGGTCTGCGAGTTACCGATGGTCCAGACGACGACCGACTGAACCCTGAGTTAGCTATTAATGCTGCCGCTAAGCAACTTGCAGATCTGGTAGGGAAGTTTGATGGCGATGAACTCAAAGCTGCCCTTGCGTACAACCAAGGCGAGGGACGCTTg                                                                                                                                                                                                                                     >  1:313548/1‑301 (MQ=255)`  
                                                                    `gTTCCAAAAGGCTGCTGATGCCAACGGGGTCTCTTATGACCTTTTACGTAAAGTCGCTTGGACAGAATCACGATTTGTGCCTACAGCAAAATCTAAGACTGGACCATTAGGCATGATGCAATTTACCAAGGCAACCGCTAAGGCCCTCGGTCTGCGAGTTACCGATGGTCCAGACGACGACCGACTGAACCCTGAGTTAGCTATTAATGCTGCCGCTAAGCAACTTGCAGATCTGGTAGGGAAGTTTGATGGCGATGAACTCAAAGCTGCCCTTGCGTACAACCAAGGCGAGGGACGCTTg                                                                                                                                                                                                                                     >  1:140441/1‑301 (MQ=255)`  
                                                                    `gTTCCAAAAGGCTGCTGATGCCAACGGGGTCTCTTATGACCTTTTACGTAAAGTCGCTTGGACAGAATCACGATTTGTGCCTACAGCAAAATCTAAGACTGGACCATTAGGCATGATGCAATTTACCAAGGCAACCGCTAAGGCCCTCGGTCTGCGAGTTACCGATGGTCCAGACGACGACCGACTGAACCCTGAGTTAGCTATTAATGCTGCCGCTAAGCAACTTGCAGATCTGGTAGGGAAGTTTGATGGCGATGAACTCAAAGCTGCCCTTGCGTACAACCAAGGCGAGGGACGCTTg                                                                                                                                                                                                                                     >  1:225155/1‑301 (MQ=255)`  
                                                                                 `gctgATTCTAACGGGTTCTCTTATGACCTTTTACGTAAAGTCGCTTGTGCAGAATCACGCTGTTTGCCTACAGCAAAATCTAAGACTGGACCATTAGGCATTATGCAATTTACCAAGGCAACCGCTAAGGCCCTCGGTCTGCGAGTTACCGATGGTCCAGACGACGACCGACTGAACCCTGATTTAGCTATTAATGCTGCCGCTAAGCAACTTGCAGGTCTGGTAGGGAAGTTTGATGGCGATGAACTCAAAGCTGCCCTTGCGTACAACCAAGGCGAGGGACGCTTGGGTAATCCACAAc                                                                                                                                                                                                                        <  1:175741/301‑1 (MQ=255)`  
                                                                                 `gctgATGCCAACGGGGTCTCTTATGACCTTTTACGTAAAGTCGCTTGGACAGAATCACGATTTGTGCCTACAGCAAAATCTAAGACTGGACCATTAGGCATGATGCAATTTACCAAGGCAACCGCTAAGGCCCTCGGTCTGCGAGTTACCGATGGTCCAGACGACGACCGACTGAACCCTGAGTTAGCTATTAATGCTGCCGCTAAGCAACTTGCAGATCTGGTAGGGAAGTTTGATGGCGATGAACTCAAAGCTGCCCTTGCGTACAACCAAGGCGAGGGACGCTTGGGTAATCCACAAc                                                                                                                                                                                                                        <  1:187296/301‑1 (MQ=255)`  
                                                                                 `gctgATGCCAACGGGGTCTCTTATGACCTTTTACGTAAAGTCGATTGGACAGAATCACGATTTGTGCCTACAGCAAAATCTAAGACTGGACCATTAGGCATGATGCAATTTACCAAGGCAACCGCTAAGGCCCTCGGTCTGCGAGTTACCGATGGTCCAGACGACGACCGACTGAACCCTGAGTTAGCTATTAATGCTGCCGCTAAGCAACTTGCAGGTCTGGTAGGGAAGTTTGATGGCGATGAACTCAAAGCTGCCCTTGCGTACAACCAAGGCGAGGGACGCTTGGGTAATCCACAAc                                                                                                                                                                                                                        >  1:183036/1‑301 (MQ=255)`  
                                                                                       `ttataagagacagctTATGACCTTTTACGTAAAGTCGCTTGGACAGGATCACGATTTGTGCCTACAGCAAAATCTAAGACTGGACCATTAGGCATGATGCAATTTACCAAGGCAACCGCTAAGGCCCTCGGTCTGCGAGTTACCGATGGTCCAGACGACGACCGACTGAACCCTGAGTTAGCTATTAATGCTGCCGCTAAGCAACTTGCAGATCTGGTAGGTAAGTTTGATGGCGATGAACTCAAAGCTGCCCTTGCGTACAAACAAGGCGAGGGACGCTTGGGTAATCCACAACTTGAgg                                                                                                                                                                                                                  <  1:154656/288‑1 (MQ=255)`  
                                                                                         `cAACGGGGTCTCTTATGACCTTTTACGTAAAGTCGCTTGGACAGAATCACGATTTGTGCCTACAGCAAAATCTAAGACTGGACCATTAGGCATGATGCAATTTACCAAGGCAACCGCTAAGGCCCTCGGTCTGCGAGTTACCGATGGTCCAGACGACGACCGACTGAACCCTGAGTTAGCTATTAATGCTGCCGCTAAGCAACTTGCAGATCTGGTAGGGAAGTTTGATGGCGATGAACTCAAAGCTGCCCTTGCGTACAACCAAGGCGAGGGACGCTTGGGTAATCCACAACTTGAGGCg                                                                                                                                                                                                                <  1:90903/301‑1 (MQ=255)`  
                                                                                              `gggTCTCTTATGACCTTTTACGGAAAGTCGCTTGGACAGAATCACGATTTGTGCCTACAGCAAAATCTAAGACTGGACCATTAGGCATGATGCAATTTACCAAGGCAACCGCTAAGGCCCTCGGTCTGCGAGTTACCGATGGTCCAGACGACGACCGACTGAACCCTGAGTTAGCTATTAATGCTGCCGCTAAGCAACTTGCAGGTCTGGTAGGGAAGTTTGATGGCGATGAACTCAAAGCTGCCCTTGCGTACAACCAAGGCGAGGGACGCTTGGGTAATCCACAACTTGAGGCGTActc                                                                                                                                                                                                           <  1:214280/301‑1 (MQ=255)`  
                                                                                               `ggTCTCTTATGACCTTTTACGTAAAGTCGCTTGGACAGAATCACGATTTGTGCCTACAGCAAAATCTAAGACTGGACCATTAGGCATGATGCAATTTACCAAGGCAACCGCTAAGGCCCTCGGTCTGCGAGTTACCGATGGTCCAGACGACGACCGACTGAACCCTGAGTTAGCTATTAATGCTGCCGCTAAGCAACTTGCAGATCTGGTAGGGAAGTTTGATGGCGATGAACTCAAAGCTGCCCTTGCGTACAACCAAGGCGAGGGACGCTTGGGTAATCCACAACTTGAGGcctgtctc                                                                                                                                                                                                          >  1:243618/1‑294 (MQ=255)`  
                                                                                                  `ctctTATGACCTTTTACGTAAAGTCGCTTGGACAGAATCACGATTTGTGCCTACAGCAAAATCTAAGACTGGACCATTAGGCATGATGCAATTTACCAAGGCAACCGCTAAGGCCCTCGGTCTGCGAGTTACCGATGGTCCAGACGACGACCGACTGAACCCTGAGTTAGCTATTAATGCTGCCGCTAAGCAACTTGCAGATCTGGTAGGGAAGTTTGATGGCGATGCACTCAAAGCTGCCCTTGCGTACAACCAAGGCGAGGGACGCTTGGGTAATCCACAACTTGAGGCGTACTCTAAg                                                                                                                                                                                                       >  1:341865/1‑301 (MQ=255)`  
                                                                                                    `tttATGACCTTTTACGTAAAGTCGCTTGGACAGAATCACGATTTGTGCCTACAGCAAAATCTAAGACTGGACCATTAGGCATGATGCAATTTACCAAGGCAACCGCTAAGGCCCTCGGTCTGCGAGTTACCGATGGTCCAGACGACGACCGACTGAACCCTGAGTTAGCTATTAATGCTGCCGCTAAGCAACTTGCAGATCTGGTAGGGAAGTTTGATGGCGATGAACTCAAAGCTGCCCTTGCGTACAACCAAGGCGAGGGACGCTTGGGTAATCCACAACTTGAGGCGTACTCTAAggg                                                                                                                                                                                                     <  1:65623/300‑1 (MQ=255)`  
                                                                                                    `ctTATTACCTTTTACGTAAGGTCGCCTTGACAGAATCACGATTTGTGCCTACAGCAAAATCTAAGCCTGGACCATTAGGCATGATGCAATTTACCAAGGCAACCGCTAAGGCCCTCGGTCTGCGAGGTACCGATTGTCCAGACGACGACCGACTGAACCCTGAGTTAGCTATTAATGCTGCCGCTAAGCAACTTGCAGATCTGGTAGTGAAGTTTGATGGCGATGAACTCAAAGCTGCCCTTGCGTACAACCAAGGCGAGGGACGCTTGGGTAATCCACAACTTGAGGCGTACTCTAAggg                                                                                                                                                                                                     <  1:256879/301‑1 (MQ=255)`  
                                                                                                    `ctTATGACCTTTTACGTAAAGTCGCTTGGACAGAATCACGATTTGTGCCTACAGCAAAATCTAAGACTGGACCATTAGGCATGATGCAATTTACCAAGGCAACCGCTAAGGCCCTCGGTCTGCGAGTTACCGATGGTCCAGACGACGACCGACTGAACCCTGAGTTAGCTATTAATGCTGCCGCTAAGCAACTTGCAGGTCTGGTAGGGAAGTTTGATGGCGATGAACTCAAAGCTGCCCTTGCGTACAACCAAGGCGAGGGACGCTTGGGTAATCCACAACTTGAGGCGTACTCTAAggg                                                                                                                                                                                                     >  1:142767/1‑301 (MQ=255)`  
                                                                                                    `ctTATGACCTTTTACGTAAAGTCGCTTGGACAGAATCACGATTTGTGCCTACAGCAAAATCTAAGACTGGACCATTAGGCATGATGCAATTTACCAAGGCAACCGCTAAGGCCCTCGGTCTGCGAGTTACCGATGGTCCAGACGACGACCGACTGAACCCTGAGTTAGCTATTAATGCTGCCGCTAAGCAACTTGCAGATCTGGTAGGGAAGTTTGATGGCGATGAACTCAAAGCTGCCCTTGCGTACAACCAAGGCGAGGGACGCTTGGGTAATCCACAACTTGAGGCGTACTCTAAggg                                                                                                                                                                                                     <  1:162712/301‑1 (MQ=255)`  
                                                                                                    `ctTATGACCTTTTACGTAAAGTCGCTTGGACAGAATCACGATTTGTGCCTACAGCAAAATCTAAGACTGGACCATTAGGCATGATGCAATTTACCAAGGCAACCGCTAAGGCCCTCGGTCTGCGAGTTACCGATGGTCCAGACGACGACCGACTGAACCCTGAGTTAGCTATTAATGCTGCCGCTAAGCAACTTGCAGATCTGGTAGGGAAGTTTGATGGCGATGAACTCAAAGCTGCCCTTGCGTACAACCAAGGCGAGGGACGCTTGGGTAATCCACAACTTGAGGCGTACTCTAAggg                                                                                                                                                                                                     <  1:94060/301‑1 (MQ=255)`  
                                                                                                    `ctTATGACCTTTTACGTAAAGTCGCTTGGACAGAATCACGATTTGTGCCTACAGCAAAATCTAAGACTGGACCATTAGGCATGATGCAATTTACCAAGGCAACCGCTAAGGCCCTCGGTCTGCGAGTTACCGATGGTCCAGACGACGACCGACTGAACCCTGAGTTAGCTATTAATGCTGCCGCTAAGCAACTTGCAGATCTGGTAGGGAAGTTTGATGGCGATGAACTCAAAGCTGCCCTTGCGTACAACCAAGGCGAGGGACGCCTGGGTAATCCACAACTTGAGGCGTACTCTAAggg                                                                                                                                                                                                     <  1:280672/301‑1 (MQ=255)`  
                                                                                                    `ctTATGACCTTTTACGTAAAGTCGCTTGGACAGAATCACGATTTGTGCCTACAGCAAAATCTAAGACTGGACCATTAGGCATGATGCAATTTACCAAGGCAACCGCTAAGGCCCTCGGTCTGCGAGTTACCGATGGTCCAGACGACGACCGACTGAACCCTGAGTTAGCTATTAATGCTGCCGCTAAGCAACTTGCAGATCTGGTAGGGAAGTTTGATGGAGATGAACTCAAAGCTGCCCTTGCGTACAACCAAGGCGAGGGACGCTTGGGTAATCCACAACTTGAGGCGTACTCTAAggg                                                                                                                                                                                                     <  1:96613/301‑1 (MQ=255)`  
                                                                                                      `tATGACCTTTTACGTAAAGTCGCTTGGACAGAATCACGATTTGTGCCTACAGCAAAATCTAAGACTGGACCAGTAGGCATGATGCAATTTACCAAGGTAACCGCTAAGGCCCTCGGTCTGCGAGTTACCGATGGTCCAGACGACGACCGACTGAACCCTGAGTTAGCTATTAATGCTGCCGCTAAGCAACTTGCAGATCTGGTAGGGAAGTTTGATGGCGATGAACTCAAAGCTGCCCTTGCGTACAACCAAGGCGAGGGACGCTTGGGTAATCCACAACTTGAGGCGTACTCTAAGGgag                                                                                                                                                                                                   <  1:34723/301‑1 (MQ=255)`  
                                                                                                        `tGACCTTTTACGTAAAGTCGCTTGGACAGAATCACGATTTGTGCCTACAGCAAAATCTAAGACTGGACCATTAGGCATGATGCAATTTACCAAGGCAACCGCTAAGGCCCTCGGTCTGCGAGTTACCGATGGTCCAGACGACGACCGACTGAACCCTGAGTTAGCTATTAATGCTGCCGCTAAGCAACTTGCAGATCTGGTAGGGAAGTTTGATGGCGATGAACTCAAAGCTGCCCTTGCGTACAACCAAGGCGAGGGACGCTTGGGTAATCCACAACTTGAGGCGTACTCTAAGGGAGAc                                                                                                                                                                                                 <  1:148345/301‑1 (MQ=255)`  
                                                                                                                 `aCGTAAAGTCGCTTGGACAGAATCACGATTTGTGCCTACAGCAAAATCTAAGACTGGACCATTAGGCATGATGCAATTTACCAAGGCAACCGCTAAGGCCCTCGGTCTGCGAGTTACCGATGGTCCAGACGATGACCGACTGAACCCTGAGTTAGCTATTAATGCTGCCGCTAAGCAACTTGCAGATCTGGTAGGGAAGTTTGATGGCGATGAACTCAAAGCTGCCCTTGCGTACAACCAAGGCGAGGGACGCTTGGGTAATCCACAACTTGAGGCGTACTCTAAgggctgtctcttctac                                                                                                                                                                                        >  1:62801/1‑288 (MQ=255)`  
                                                                                                                    `tAAAGTCGCTTGGACAGAATCACGATTTGTGCCTACAGCAAAATCTAAGACTGGACCATTAGGCATGATGCAATTTACCAAGGCAACCGCTAAGGCCCTCGGTCTGCGAGTTACCGATGGTCCAGACGACGACCGACTGAACCCTGAGTTAGCTATTAATGCTGCCGCTAAGCAACTTGCAGATCTGGTAGGGAAGTTTGATGGCGATGAACTCAAAGCTGCCCTTGCGTACAACCAAGGCGAGGGACGCTTGGGTAATCCACAACTTGAGGCGTACTCTAAGGGAGACTTCGCATCAAtc                                                                                                                                                                                     <  1:120587/301‑1 (MQ=255)`  
                                                                                                                                    `gAATCACGATTTGTGCCTACAGCAAATTCTAAGACTGGACCATTAGGCATGATGCAATTTACCAAGGCAACCGCTAAGGCCCTCGGTCTGCGAGTTACCGATGGTCCAGACGACGAACGACTGAACCCTGAGTTAGCTATTAATGCTGCCGCTAATCAACTTGCAGATCTGGTAGGGAAGTTTGATGGCGATGAACTCAAAGCTGCCCTTGCGTACAACAAAGGCGAGGGACGCTTGGGTAATCCACAACTTGTGGCGTACTCTAAGGGAGACTTCGCCTCACTCTCTGAGGAGGGACGTa                                                                                                                                                                     >  1:123445/1‑301 (MQ=255)`  
                                                                                                                                    `gAATCACGATTTGTGCCTACAGCAAAATCTAAGACTGGACCATTAGGCATGATGCAATTTACCAAGGCAACCGCTAAGGCCCTCGGTCTGCGAGTTACCGATGGTCCAGACGACGACCGACTGAACCCTGAGTTAGCTATTAATGCTGCCGCTAAGCAACTTGCAGGTCTGGTAGGGAAGTTTGATGGCGATGAACTCAAAGCTGCCCTTGCGTACAACCAAGGCGAGGGACGCTTGGGTAATCCACAACTTGAGGCGTACTCTAAGGGAGACTTCGCATCAATCTCTGAGGAGGGACGTa                                                                                                                                                                     >  1:217721/1‑301 (MQ=255)`  
                                                                                                                                     `ataagagacaggtgCCTACAGCAAACGCTGAGACTTGACCATTGAGCAAGATGGAATTTATCCAGGCAACCGCTAAGGGCCTCGGTCTGCGAGTTAACGATGGTCCAGACGACGACCGACTGAACCCTGATTTAGCTATTAATGCTGCCGCTAAGCAACTTGCAGTTATGGTAGGGAAGTTTGATGGCGATGAACTCAAAGCTGCCCTTGCGTACAACCAAGGCGAGGGACGCTT‑GGTAATCCACAACTTGAGGCGTACTCTAAGGGAGACTTCGCATCAATCTCTGAGGAGGGACGTAAc                                                                                                                                                                   <  1:344789/290‑1 (MQ=255)`  
                                                                                                                                      `aTCACGATTTGTGCCTACAGCAAAATCTAAGACTGGACCATTAGGCATGATGCAATTTACCAAAGCAACCGCTAATGCCCTCGGTCTACGAGTTACCGATGTTCCAGCCGACGACCGACTGAACCCTGAGTTAGCTATTAATGCTGCCGCTAAGCACATTTCAGATCTGGTAGGGAAATTTGATGGCTATGAACTCAAAACTTCCCTTGCTTACAACCAAGTCACGTGCCGCTTGGGTAATCCACACCTTACGGCGTACTCTAAGGCAGACTTCGCATCAATCTCTGATGCGGGActtcac                                                                                                                                                                   >  1:146/1‑296 (MQ=255)`  
                                                                                                                                        `cACGATTTGTGCCTACAGCAAAATCTAAGACTGGACCATTAGGCATGATGCAATTTACCAAGGCAACCGCTAAGGCCCTCGGTCTGCGAGTTACCGATGGTCCAGACGACGACCGACTGAACCCTGAGTTAGCTATTAATGCTGCCGCTAAGCAACTTGCAGATCTGGTAGGGAAGTTTGATGGCGATGAACTCAAAGCTGCCCTTGCGTACAACCAAGGCGAGGGACGCTTGGGTAATCCACAACTTGAGGCGTACTCTAAGGGAGACTTCGCATCAATCTCTGAGGAGGGACGTAACTa                                                                                                                                                                 >  1:319325/1‑301 (MQ=255)`  
                                                                                                                                          `cGATTTGTGCCTACAGCAAAATCTAAGACTGGACCATTAGGCATGATGCAATTTACCAAGGCAACCGCTAAGGCCCTCGGTCTGCGAGTTACCGATGGTCCAGACGACGACCGACTGAACCCTGAGTTAGCTATTAATGCTGCCGCTAAGCAACTTGCAGATCTGGTAGGGAAGTTTGATGGCGATGAACTCAAAGCTGCCCTTGCGTACAACCAAGGCTAGGGACGCTTGGGTAATCCACAACTTGAGGCGTACTCTAAGGGAGACTTCGCATCAATCTCTGAGGAGGGACGTAACTACa                                                                                                                                                               <  1:196370/301‑1 (MQ=255)`  
                                                                                                                                              `ttGTGCCTACAGCAAAATCTAAGACTGGACCATTAGGCATGATGCAATTTACCAAGGCAACCGCTAAGGCCCTCGGTCTGCGAGTTACCGATGGTCCAGACGACGACCGACTGAACCCTGAGTTAGCTATTAATGCTGCCGCTAAGCAACTTGCAGATCTGGTAGGGAAGTTTGATGGCGATGAACTCAAAGCTGCCCTTGCGTACAACCAAGGCGAGGGACGCTTGGGTAATCCACAACTTGAGGCGTACTCTAAGGGAGACTTCGCATCAATCTCTGAGGAGGGACGTAACTACATGCg                                                                                                                                                           <  1:170377/301‑1 (MQ=255)`  
                                                                                                                                                `gtgCCTACAGCAAAATCTAAGACTGGACCATTAGGCATGATGCAATTTACCAAGGCAACCGCTAAGGCCCTCGGTCTGCGAGTTACCGATGGTCCAGACGACGACCGACTGAACCCTGAGTTAGCTATTAATGCTGCCGCTAAGCAACTTGCAGATCTGGTAGGGAAGTTTGATGGCGATGAACTCAAAGCTTCCCTTGCGTACAACCAAGGCGAGGGACGCTTGGGTAATCCACAACTTGAGCCGTACTCTAAGGGTGACTTCGCATCAATCTCTGAGGAGGGTCGTAACTACATGCGTa                                                                                                                                                         >  1:231526/1‑301 (MQ=255)`  
                                                                                                                                                  `aggtgtataagcgacagaGACTGGACCATTAGGCATGATGCAATTTACCAAGGCAACCGCTAAGGCCCTCGGTCTGCGAGTTACCGATGGTCCAGACGACGACCGACTGAACCCTGAGTTAGCTATTAATGCTGCCGCTAAGCAACTTGCAGATCTGGTAGGGAAGTTTGATGGCGATGAACTCAAAGCTGCCCTTGCGTACAACCAAGGCGAGGGACGCTTGGGTAATCCACAACTTGAGGCGTACTCTAAGGGAGACTTCGCATCAATCTCTGAGGAGGGACGTAACTACATGCGTAAc                                                                                                                                                       <  1:70178/284‑1 (MQ=255)`  
                                                                                                                                                    `aagatactgaaTCTCAGCCTTGACCATTAGGCATGTGGCAATTTACCAAGGCAACCGCTAATGCCCTCGGTCTGTGCGTTACCGATGGTCCAGACGACGACCGACTGAACCCTGAGTTAGCTATTATTGCTGCCGCTAAGCAACTTGCAGGTCTGGTAGGGAAGTTTGATGGCGATGAACTCAAAGCGGCCCTTGCGTACAACCAAGGCGAGGGACGCTTGGGTAATCCACAACTTGAGGCGTACTCTAAGGGAGACTTCGCATCAATCTCTGAGGAGGGACGTAACTACATGCGTAACCt                                                                                                                                                     <  1:315731/292‑1 (MQ=255)`  
                                                                                                                                                           `agagacagaGACTGGACCATTAGGCATGATGCAATTTACCAAGGCAACCGCTAAGGCCCTCGGTCTGCGAGTTACCGATGGTCCAGACGACGACCGACTGAACCCTGAGTTAGCTATTAATGCTGCCGCTAAGCAACTTGCAGGTCTGGTAGGGAAGTTTGATGGCGATGAACTCAAAGCTGCCCTTGCGTACAACCAAGGCGAGGGACGCTTGGGTAATCCACAACTTGAGGCGTACTCTAAGGGAGACTTCGCATCAATCTCTGAGGAGGGACGTAACTACATGCGTAACCTTCTGGAt                                                                                                                                              <  1:228380/293‑1 (MQ=255)`  
                                                                                                                                                              `aTCTAAGACTGGACCATTAGGCATGATGCAATTTACCAAGGCAACCGCTAAGGCCCTCGGTCTGCGAGTTACCGATGGTCCAGACGACGACCAACTGAACCCTGAGTTAGCTATTAATGCTGCCGCTAAGCAACTTGCAGATCTGGTAGGGAAGTTTGATGGCGATGAACTCAAAGCTGCCCTTGCGTACAACCAAGGCGAGGGACGCTTGGGTAATCCACAACTTGAGGCGTACTCTACGGGAGACTTCGCATCAATCCCTGAGGAGGGACGTAACTACATGCGTAACCTTCTGGATGtt                                                                                                                                           >  1:204812/1‑301 (MQ=255)`  
                                                                                                                                                                   `aGACTGGACCATTAGGCATGATGCAATTTACCAAGGCAACCGCTAAGGCCCTCGGTCTGCGAGTTACCGATGGTCCAGACGACGACCGACTGAACCCTGAGTTAGCTATTAATGCTGCCGCTAAGCAACTTGCTGATCTGGTAGGGAAGTTTGATGGCGATGAACTCAACGCTGCCCTTGCGTACAACCACGGCGAGGGACTCTTGGGTACTCCACAACTTGAGTCGTACTCTAAGGGAGACTTCGCATCAATCTCTGAGGAGGGACGTACCTACATTCGTAACCTTCTGGATGTTGCCaa                                                                                                                                      >  1:336390/1‑301 (MQ=255)`  
                                                                                                                                                                    `gACTTGACCATTAGGCATGATGCAATTTACCAAGGCAACCGCTAAGGCCCTCGGTCTGCGAGTTACCGATGGTCCAGACGACGACCGACTGAACCCTGAGTTAGCTATTAATGCTGCCGCTAAGCAACTTGCAGATCTGGTAGGGAAGTTTGATGGCGATGAACTCAAAGCTGCCCTTGCGTACAACCAAGGCGAGGGACGCTTGGGTAATCCACAACTTGAGGCGTACTCTAAGGGAGACTTCGCATCAATCTCTGAGGAGGGACGTAACTACATGCGTAACCTTCTGGATGTTGCTAAg                                                                                                                                     >  1:107005/1‑301 (MQ=255)`  
                                                                                                                                                                         `gACCATTAGGCATGATGCAATTTACCAAGGCAACCGCTAAGGCCCTCGGTCTGCGAGTTACCGATGGTACAGACGACGACCGACTGAACCCTGAGTTAGCTCTTAATGCTGCCGCTAAGCAACTTGCCGATCTGGTCGGGAAGTTTGATGTCGATGAACTCAACGCTGCCCTTGCGTACAACCATGGCGAGGGACGCTTGGGTAATCCACAACTTGAGGCAGACTCTAAGGGAGACTTCGCATCAATCTCTGAGGAAGGATGTAACTCCATGCGTAACCATCTGGACGTTTCTAAGTTAcc                                                                                                                                >  1:75330/1‑301 (MQ=255)`  
                                                                                                                                                                          `cagaTTAGGCATGATGCAAATTACCAAGGCAACCGCTAAGGCCCTCGGTCTGCGAGTTACCGATGGTCCAGACGACGACCGACTGAACACTGAGTTAGCTATTAATGCTGCCGCTAAGCAACTTGCAGATCTGGTAGGGAAGTTTGATGGCGATGAACTCAAAGCTGCCCTTGCGTACAACCAAGGCGAGGGACGCTTGGGTAATCCACAACTTGAGGCGTACTCTAAGGGAGACTTCGCATCAATCTCTGAGGAGGGACGTAACTACATGCGTAACCTTCTGGATGTTGCTAAGTCACCt                                                                                                                               <  1:59617/298‑1 (MQ=255)`  
                                                                                                                                                                            `cATTAGGCATGATGCAATTTACCAAGGCAACCGCTAAGGCCCTCGGTCTGCGAGTTACCGATGGTCCAGACGACGACCGACTGAACCCTGAGTTAGCTATTAATGCTGCCGCTAAGCAACTTGCAGATCTGGTAGGGAAGTTTGATGGCGATGAACTCAAAGCTGCCCTTGCGTACAACCAAGGCGAGGGACGCTTGGGTAATCCACAACTTGAGGCGTACTCTAAGGGAGACTTCGCATCAATCTCTGAGGAGGGACGTAACTACATGCGTAACCTTCTGGATGTTGCTAAGTCACCTAt                                                                                                                             <  1:293497/301‑1 (MQ=255)`  
                                                                                                                                                                               `tAGGCATGATGCAATTTGCCAAGGCAACCGCTAAGGCCCTCGGTCTGCGAGTTACCGATGGTCCAGACGACGACCGACTGAACCCTGAGTTAGCTATTAATGCTGCCGCTAAGCAACTTGCAGATCTGGTAGGGAAGTTTGATGGCGATGAACTCAAAGCTGCACTTGCGTACAACCAAGGCGAGGGACGCTTGGGTAATCCACAACTTGAGGCGTACTCTAAGGGAGACTTCGCATCAATCTCTGAGGAGGGACGTAACTACATGCGTAACCTTCTGGATGTTGCTAAGTCACCTATgac                                                                                                                          <  1:299092/301‑3 (MQ=255)`  
                                                                                                                                                                                     `tgatgCAATTTACCAAGGCAACCGCTAAGGCCCTCGGTCTGCGAGTTACCGATGGTCCAGACGACGACCGACTGAACCCTGAGTTAGCTATTAATGCTGCCGCTAAGCAACTTGCAGATCTGGTAGGGAAGTTTGATGGCGATGAACTCAAAGCTGCCCTTGCGTACAACCAAGGCGAGGGACGCTTGGGTAATCCACAACTTGAGGCGTACTCTAAGGGAGACTTCGCATCAATCTCTGAGGAGGGACGTAACTACATGCGTAACCTTCTGGATGTTGCTAAGTCACCTATGACTGGAcc                                                                                                                    >  1:46377/1‑300 (MQ=255)`  
                                                                                                                                                                                       `atgCAATTTACCAAGGCAACCGCTAAGGCCCTCGGTCTGCGAGTTACCGATGGTCCAGACGACGACCGACTGAACCCTGAGTTAGCTATTAATGCTGCCGCTAAGCAACTTGCAGATCTGGTAGGGAAGTTTGATGGCGATGAACTCAAAGCTGCCCTTGCGTACAACCGAGGCGAGGGACGCTTGGGTAATCCACAACTTGAGGAGTACTCTAAGGGAGACTTCGCATCCATCTCTGAGGCGGGACGTAACTACATGCGTAACCTTCTGGATGTTGCTAAGTCACCTATGGCTGGACAgc                                                                                                                  >  1:28912/1‑300 (MQ=255)`  
                                                                                                                                                                                              `ttACCAAGGCAACCGCTAAGGCCCTCGGTCTGCGAGTTACCGATGGTCCAGACGACGACCGACTGAACCCTGAGTTAGCTATTAATGCTGCCGCTAAGCAACTTGCAGGTCTGGTAGGGAAGTTTGATGGCGATGAACTCAAAGCTGCCCTTGCGTACAACCAAGGCGAGGGACGCTTGGGTAATCCACAACTTGAGGCGTACTCTAAGGGAGACTTCGCATCAATCTCTGAGGAGGGACGTAACTACATGCGTAACCTTCTGGATGTTGCTAAGTCACCTATGGCTGGACAGTTGGAAAc                                                                                                           <  1:274339/301‑1 (MQ=255)`  
                                                                                                                                                                                              `tgaCCAAGGCAACCACGAAGGCCCTCGGTCTTCGAGTTACCGATGGTCCAGACGACGACCGACTGAACCATGAGTTAGCTATTAATGCTGCCGCTAAGCAACTTGCAGATCTGGTAGGGAAGTTTGATGGCGATGAACTCAAAGCTGCCCTTGCGTACAACCAAGGCGAGGGACGCTTGGGTAATCCACAACTTGAGTCGTACTCTAAGGGAGACTTCGCATCAATCTCTGAGGAGGGACGTAACTACATGCGTAACCTTCTGGATGTTGCTAAGTCACCTATGACTGGACAGTTGGAAAc                                                                                                           <  1:30952/299‑1 (MQ=255)`  
                                                                                                                                                                                                                 `ggCCCTCGGTCTGCGAGTTACCGATGGTCCAGACGACGACCGACTGAACCCTGAGTTAGCTATTAATGCTGCCGCTAAGCAACTTGCAGATCTGGTAGGGAAGTTTGATGGCGATGAACTCAAAGCTGCCCTTGCGTACAACCAAGGCGAGGGACGCTTGGGTAATCCACAACTTGAGGCGTACTCTAAGGGAGACTTCGCATCAATCTCTGAAGAGGGACGTAACTACATGCGTAACCTTCTGGATGTTGCTAAGTCACCTATGGCTGGACAGTTGGAAACTTTTGGTGGCATAACCCCa                                                                                        >  1:322244/1‑301 (MQ=255)`  
                                                                                                                                                                                                                    `ccTCGGTCTGCGAGTTACCGATGGTCCAGACGACGACCGACTGAACCCTGAGTTAGCTATTAATGCTGCCGCTAAGCAACTTGCAGATCTGGTAGGGAAGTTTGATGGCGATGAACTCAAAGCTGCCCTTGCGTACAACCAAGGCGAGGGACGCTTGGGTAATCCACAACTTGAGGCGTACTCTAAGGGAGACTTCGCATCCATCTCTGAGGAGGGACGTAACTACATGCGTAACCTTCTGGATGTTGCTAAGTCACCTATGGCTGGACAGTTGGAAACTTTTGGTGGCATAACCCCAAAg                                                                                     <  1:40249/301‑1 (MQ=255)`  
                                                                                                                                                                                                                     `cTCGGTCTGCGAGTTACCGATGGTCCAGACGACGACCGACTGAACCCTGAGTTAGCTATTAATGCTGCCGCTAAGCAACTTGCAGATCTGGAAGGGAAGTTTGATGGCGATGAACTCAACGCTGACCTTGCGTACAAACAAGGCGAGGGACGCTTGGGTAATCCACAACTTGAGGCGTACTCTAAGGAAGACTTCGCATCAATCTCTGAGGAGGGACGTAACTACATGCGTAACCTTCTGGATGTTGCTAAGTCACCTATGGCTGGACAGTTGGAAACTTTTGGTGGCATAACCCCAAAgg                                                                                    >  1:272813/1‑301 (MQ=255)`  
                                                                                                                                                                                                                         `gTCTGCGAGTTACCGATGGTCCAGACGACGACCGACTGAACCCTGAGTTAGCTATTAATGCTGCCGCTAAGCAACTTGCAGATCTGGTAGGGAAGTTTGATGGCGATGAACTCAAAGCTGCCCTTGCGTACAACCAAGGCGAGGGACGCTTGGGTAATCCACAACTTGAGGCGTACTCTAAGGGAGACTTCGCATCAATCTCTGAGGAGGGACGTAACTACATGCGTAACCTTCTCGATGTTGCTAAGTCACCTATGACTGGACAGTTGGAAACTTTTGGTGGCATAACCCCAAAGGGTaa                                                                                >  1:302850/1‑301 (MQ=255)`  
                                                                                                                                                                                                                             `gCGAGTTACCGATGGTCCAGACGACGACCGACTGAACCCTGAGTTAGCTATTAATGCTGCCGCTAAGCAACTTGCAGGTCTGGTAGGGAAGTTTGATGGCGATGAACTCAAAGCTGCCCTTGCGTACAACCAAGGCGAGGGACGCTTGGGTAATCCACAACTTGAGGCGTACTCTAAGGGAGACTTCGCATCAATCTCTGAGGAGGGACGTAACTACATGCGTAACCTTCTGGATGTTGCTAAGTCACCTATGGCTGGACAGTTGGAAACTTTTGGTGGCATAACCCCAAAGGGTAAAGGc                                                                            >  1:254463/1‑301 (MQ=255)`  
                                                                                                                                                                                                                             `gCGAGTTACCGATGGTCCAGACGACGACCGACTGAACCCTGAGTTAGCTATTAATGCTGCCGCTAAGCAACTTGCAGATCTGGTAGGGAAGTTTGATGGCGATGAACTCAAAGCTGCCCTTGCGTACAACCAAGGCGAGGGACGCTTGGGTAATCCACAACTTGAGGCGTACTCTAAGGGAGACTTCGCATCAATCTCTGAGGAGGGACGTAACTACATGCGTAACCTTCTGGATGTTGCTAAGTCACCTATGGCTGGACAGTTGGAAACTTTTGGTGGCATAACCCCAAAGGGTAAAGGc                                                                            >  1:325778/1‑301 (MQ=255)`  
                                                                                                                                                                                                                                `aGTTACCGATGGTCCAGACGACGACCGACTGAACCCTGAGTTAGCTATTAATGCTGCCGCTAAGCAACTTGCAGATCTGGTAGGGCAGTTTGATGGCGATGAACTCAAAGCTGCCCTTGCGTACAACCAAGGCGCGGGCCGCTTGGGTAATCCACAACTTGAGGCGTACTATAAGGGAGACTTCGCATCAATCTCTGAGGAGGGACGTAACTACATGCGTAACCTTCTGGATGTTGCTAAGTCACCTATGGCTGGACAGTTGGAAACTTTTGGTGGCATACACCCAAAGGGTAAAGGCAtt                                                                         >  1:339216/1‑301 (MQ=255)`  
                                                                                                                                                                                                                                                `gacgacgacCGACTGAACCCTGAGTTAGCTATTAATGCTGCCGCTAAGCAACTTGCAGATCTGGTAGGGAAGTTTGATGGCGATGAACTCAAAGCTGCCCTTGCGTACAACCAAGGCGAGGGACGCTTGGGTAATCCACAACTTGAGGCGTACTCTAAGGGAGACTTCGCATCAATCTCTGAGGAGGGACGTAACTACATGCGTAACCTTCTGGATGTTGCTAAGTCACCTATGGCTGGACAGTTGGAAACTTTTGGTGGCATAACCCCAAAGGGTAAAGGCATTCCGGCTGAGGTAGGAt                                                         <  1:134365/301‑1 (MQ=255)`  
                                                                                                                                                                                                                                                    `acgacCGACTGAACCCTGAGTTAGCTATTAATGCTGCCGCTAAGCAACTTGCAGATCTGGGAGGGAAGTTTGATGGCGATGAACTCAAAGCTGCCCTTGCGTACAACCAAGGCGAGGGACGCTTGGGTAATCCACAACTTGAGGCGTACTCTAAGGGAGACTTCGCATCAATCTCTGAGGAGGGACGTAACTACATGCGTAACCTTCTGGATGTTGCTAAGTCACCTATGGCTGGACAGTTGGAAACTTTTGGTGGCATAACCCCAAAGGGTAAAGGCATTCCGGCTGTGGTAGGATTggt                                                     >  1:233896/1‑300 (MQ=255)`  
                                                                                                                                                                                                                                                        `ccgacTGAACCCTGAGTTAGCTATTAATGCTGCCGCTAAGCAACTTGCAGATCTGGTAGGGAAGTTTGATGGCGATGAACTCAAAGCTGCCCTTGCGTACAACCAAGGCGAGGGACGCTTGGGTAATCCACAACTTGAGGCGTACTCTAAGGGAGACTTCGCATCAATCTCTGAGGAGGGACGTAACTACATGCGTAACCTTCTGGATGTTGCTAAGTCACCTATGGCTGGACAGTTGGAAACTTTTGGTGGCATAACCCCAAAGGGTAAAGGCATTCCGGCTGAGGTAGGATTGGCTGGa                                                 >  1:56009/1‑301 (MQ=255)`  
                                                                                                                                                                                                                                                          `gacTGAACCCTGAGTTAGCTATTAATGCTGCCGCTAAGCAACTTGCAGATCTGGTAGGGAAGTTTGATGGCGATGAACTCAAAGCTGCCCTTGCGTACAACCAAGGCGAGGGACGCTTGGGTAATCCACAACTTGAGGCGTACTCTAAGGGAGACTTCGCATCAATCTCTGAGGAGGGACGTAACTACATGCGTAACCTTCTGGATGTTGCTAAGTCACCTATGGCTGGACAGTTGGAAACTTTTGGTGGCATAACCCCAAAGGGTAAAGGCATTCCGGCTGAGGTAGGATTGGCTGGAAt                                               <  1:42271/301‑1 (MQ=255)`  
                                                                                                                                                                                                                                                           `acTGAACCCTGAGTTAGCTATTAATGCTGCCGCTAAGCAACTTGCAGATCTGGTAGGGAAGGTTGATGGCGATGAACTCAAAGCTGCCCTTGCGTACAACCAAGGCGAGGGACGCTTGGGTAATCCACAACTTGAGGCGTACTCTAAGGGAGACTTCGCATCAATCTCTGAGGAGGGACGTAACTACATGCGTAACCTTCTGGATGTTGCTAAGTCACCTATGGCTGGACAGTTGGAAACTTTTGGTGGCATAACCCCAAAGGGTAAAGGCATTCCGGCTGAGGTAGGATTGGCTGGAAtt                                              <  1:262581/301‑1 (MQ=255)`  
                                                                                                                                                                                                                                                               `agcccTGAGTTAGCTATTAATGCTGCCGCTAAGCAACTTGCAGATCTGGTAGGGAAGTTTGATGGCGATGAACTCAAAGCTGCCCTTGCGTACAACCAAGGCGAGGGACGCTTGGGTAATCCACAACTTGAGGCGTACTCTAAGGGAGACTTCGCATCAATCTCTGAGGAGGGACGTAACTACATGCGTAACCTTCTGGATGTTGCTAAGTCACCTATGGCTGGACAGTTGGAAACTTTTGGTGGCATAACCCCAAAGGGTAAAGGCATTCCGGCTGAGGTAGGATTGGCTGGAATTGGTc                                          <  1:23670/299‑1 (MQ=255)`  
                                                                                                                                                                                                                                                                              `aTTAATGCTGCCGCTAAGCAACTTGCAGGTCTGGTAGGGAAGTTTGATGGCTCTGACCTCACAGCTTCCCTTTCTTACAACCAAGTCTTTTTACGCTTGTTTAATCCACAACTTTCTCCTTACTCTAATTCTCTCTTCTCATCCTTCTCTGAGGATTGACCTAACTACATGCTTTCCCTTCTGGCTGTTTCTAAGTCACCTATGGCTCGTCAGTTGCATACTTTTGTTTTCTTAACCCCAATTGGTATATGCATTCCGGCTGTGGTAGGTTTGGCTGGACTTGGTCACTAGCAgttcgttt                           >  1:166647/1‑294 (MQ=255)`  
                                                                                                                                                                                                                                                                               `ttAATGCTGCCGCTAAGCAACTTTCAGATCTGGTAGGGAAGTTTGATGGCGATGTACTCAAAGCTGCCCTTGCGTACAACCAAGTCGAGGGACGCTTGGGTAATCCACAACTTGAGGCGTACTCTAAGAGAGACTTCGCATCAATCTCTGAGGAGGGACGTAACTACATGCGTAACCTTCTGGATGTTGCTAAGTCACCTATGGCTGGACAGTTGGAAACTTTTGGTGGCATAACCCCAAAGGGTAAAGGCATTCCGGCTGAGGTAGGATTGGCTGGAATTGGTCACAAGCAGAAAGTAac                          <  1:189488/301‑1 (MQ=255)`  
                                                                                                                                                                                                                                                                               `ttAATGCGGCCGCTAAGCAACTTGCAGATCTGGTAGGGAAGTTTGATGGCGATGAACTAAAAGCTGCCCTTGCGTACAACCAAGGCGAGGGACGCTTGGGTAATCCACAACTTGAGGCGTACTCTAAGGGAGACTTCGCATCAATCTCTGAGGAGGGACGTAACTACATGCGTAACCTTCTGGATGTTGCTAAGTCACCTATGGCTGGACAGTTGGAAACTTTTGGTGGCATAACCCCAAAGGGTAAAGGCATTCCGGCTGAGGTAGGATTGGCTGGAATTGGTCACAAGCAGAAAGTAac                          <  1:127654/301‑1 (MQ=255)`  
                                                                                                                                                                                                                                                                                 `aaTGCTGCCGCTAAGCAACTTGCAGGTCTGGTAGGGAAGTTTGATGGCGATGAACTCAAAGCTGCCCTTGCGTACAACCAAGGCGAGGGACGCTTGGGTAATCCACAACTTGAGGCGTACTCTAAGGGAGACTTCGCATCAATCTCTGAGGAGGGACGTAACTACATGCGTAACCTTCTGGATGTTGCTAAGTCACCTATGGCTGGACAGTTGGAAACTTTTGGTGGCATAACCCCAAAGGGTAAAGGCATTCCGGCTGAGGTAGGATTGGCTGGAATTGGTCACAAGCAGAAAGTAacac                        <  1:291541/301‑1 (MQ=255)`  
                                                                                                                                                                                                                                                                                           `cTAAGCAACTTGCAGATCTGGTAGGGAAGTTTGATGGCGATGAACTCAAAGCTGCCCTTGCGTACAACCAAGGCGAGGGACGCTTGGGTAATCCACAACTTGAGGCGTACTCTAAGGGAGACTTCGCATCAATCTCTGAGGAGGGACGTAACTACATGCGTAACCTTCTGGATGTTGCTAAGTCACCTATGGCTGGACAGTTGGAAACTTTTGGTGGCATAACCCCAAAGGGTAAAGGCATTCCGGCTGAGGTAGGATTGGCTGGAATTGGTCACAAGCAGAAAGTAACACAGGAACTTcc              <  1:100148/301‑1 (MQ=255)`  
                                                                                                                                                                                                                                                                                                       `gaGGTCTGGTAGGGAAGTTTGAGGGCGATGAACTCAAAGCTGCCCTTGCGTACAACCAAGGCGAGGGACGCTTGGGTAATCCACAACTTGAGGCGTACTCTAAGGGAGACTTCGCATCAATCTCTGAGGAGGGACGTAACTACATGCGTAACCTTCTGGATGTTGCTAAGTCACCTATGGCTGGACAGTTGGAAACTTTGGGTGGCATAACCACAAAGGGTAAAGGCATTCCGGCTGAGGTAGGATTGGCTGGAATTGGTCACAAGCAGAAAGTAACACAGGAACTTCCTGAGTCCACAAg  <  1:20198/300‑1 (MQ=255)`  

                                                                                                                                                                                                                                                                                                          `|`                                                                                                                                                                                                                                                                                                           
`AAAGGCTACATAAGGAGGCCCTAAATGGATAAGTACGATAAGAACGTACCAAGTGATTATGATGGTCTGTTCCAAAAGGCTGCTGATGCCAACGGGGTCTCTTATGACCTTTTACGTAAAGTCGCTTGGACAGAATCACGATTTGTGCCTACAGCAAAATCTAAGACTGGACCATTAGGCATGATGCAATTTACCAAGGCAACCGCTAAGGCCCTCGGTCTGCGAGTTACCGATGGTCCAGACGACGACCGACTGAACCCTGAGTTAGCTATTAATGCTGCCGCTAAGCAACTTGCAGGTCTGGTAGGGAAGTTTGATGGCGATGAACTCAAAGCTGCCCTTGCGTACAACCAAGGCGAGGGACGCTTGGGTAATCCACAACTTGAGGCGTACTCTAAGGGAGACTTCGCATCAATCTCTGAGGAGGGACGTAACTACATGCGTAACCTTCTGGATGTTGCTAAGTCACCTATGGCTGGACAGTTGGAAACTTTTGGTGGCATAACCCCAAAGGGTAAAGGCATTCCGGCTGAGGTAGGATTGGCTGGAATTGGTCACAAGCAGAAAGTAACACAGGAACTTCCTGAGTCCACAAG  >  NC_001604.1/30571‑31166`

  

Alignment Legend

Aligned base mismatch/match (shaded by quality score): `ATCG/ATCG` `< 3 ≤` `ATCG/ATCG` `< 10 ≤` `ATCG/ATCG` `< 22 ≤` `ATCG/ATCG` `< 34 ≤` `ATCG/ATCG` `< 38 ≤` `ATCG/ATCG`

Unaligned base: `atcg`    Masked matching base: `atcg`    Alignment gap: `‑`    Deleted base: `‑`
